# Sleep and Motor Learning: Implications for Physical Rehabilitation After Stroke

**DOI:** 10.3389/fneur.2015.00241

**Published:** 2015-11-24

**Authors:** Christel Gudberg, Heidi Johansen-Berg

**Affiliations:** ^1^Oxford Centre for Functional MRI of the Brain (FMRIB), Nuffield Department of Clinical Neurosciences, University of Oxford, John Radcliffe Hospital, Oxford, UK; ^2^Sleep and Circadian Neuroscience Institute (SCNi), Nuffield Department of Clinical Neurosciences, University of Oxford, Sir William Dunn School of Pathology, Oxford, UK

**Keywords:** sleep, motor memory, consolidation, plasticity and learning, rehabilitation, motor recovery, stroke, aging

## Abstract

Sleep is essential for healthy brain function and plasticity underlying learning and memory. In the context of physical impairment such as following a stroke, sleep may be particularly important for supporting critical recovery of motor function through similar processes of reorganization in the brain. Despite a link between stroke and poor sleep, current approaches to rehabilitative care often neglect the importance of sleep in clinical assessment and treatment. This review assimilates current evidence on the role of sleep in motor learning, with a focus on the implications for physical rehabilitation after stroke. We further outline practical considerations for integrating sleep assessment as a vital part of clinical care.

## Introduction

The adult brain is highly adaptable, even after injury it often exhibits an impressive capacity for reorganization. Activity in the brain during sleep is thought to be critically involved in supporting these processes of plasticity. Briefly, sleep can be thought of as a state of consciousness, or alternations in consciousness, which oscillates between states of reduced awareness of external real-world stimuli to a complete loss of consciousness (1). While the precise mechanisms have yet to be clearly defined, sleep has been associated with many important functions, including those of the immune and memory systems (2–5). In memory, sleep is consistently attributed a particularly prominent role in supporting time-sensitive processes associated with the consolidation of memories. Consolidation here refers to dynamic processes in the brain that occur after initial (“on-line”) memory encoding takes place, such as when we practice a new skill. Subsequent (“off-line”) mechanisms of consolidation serve to further process these new memory traces, for instance, to enable the integration of knowledge and long-term memory storage.

One reason memory consolidation may be particularly important in a clinical context is because of how it applies to neurological rehabilitation, such as motor recovery after lesion to the brain. Here, the primary aim of physical rehabilitation is to facilitate recovery of functional motor capacity after initial impairment. Another way to look at physical rehabilitation, therefore, is as a form of motor learning, or relearning, which in turn may tap into some of the same processes of memory formation and consolidation as other forms of procedural memory (6, 7). Consequently, experimental insights into processes in the brain that support motor memory are likely to have more wide-ranging application that may benefit understanding and development of useful strategies for improving long-term rehabilitative outcomes in the clinic. The primary objective of this review is to provide an assimilation of current evidence on the role of sleep in motor learning and to identify specific factors of learning and consolidation that may have important implications for rehabilitation. For the purposes of this review, we will focus primarily on sleep-dependent motor memory with relevance to physical rehabilitation after stroke, although many of the discussion points included here will likely apply more broadly to other types of memory and rehabilitation. Meanwhile, what is some of the evidence linking sleep, in particular, to motor memory?

## Sleep and Motor Learning

**Figure 1:**
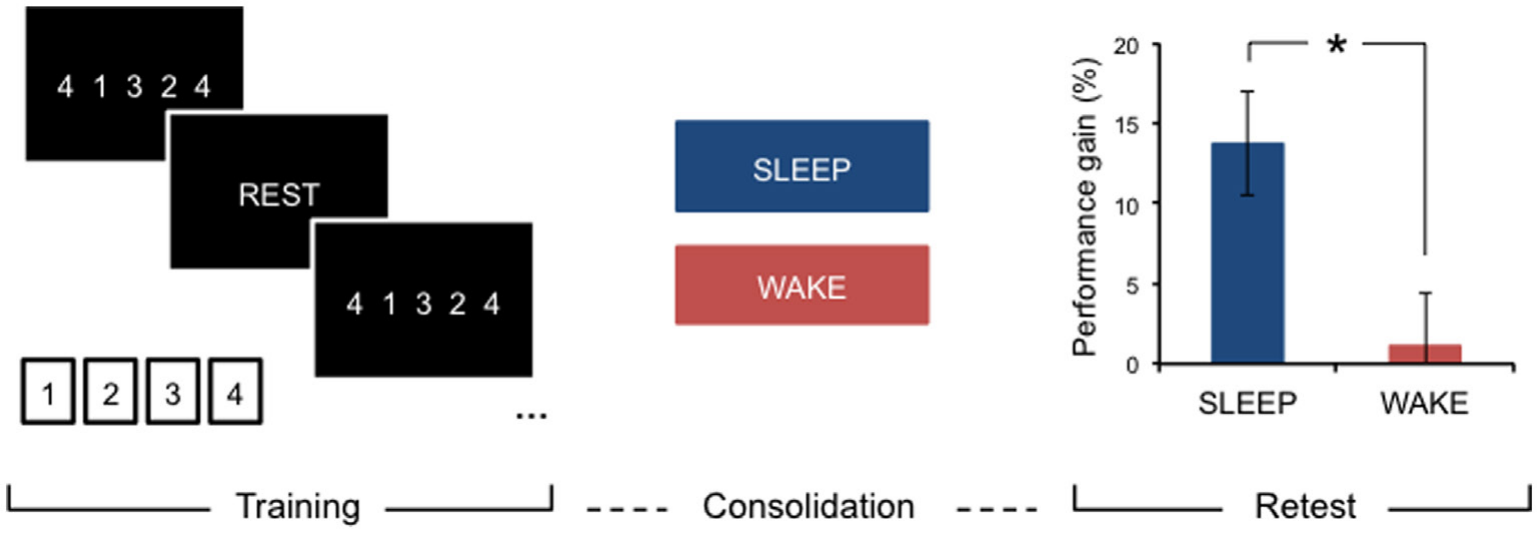
**Motor learning and sleep-dependent consolidation**. After a period of consolidation following training on an explicit motor sequence-learning task performed on a standard keyboard or button box, young, healthy adults consistently demonstrate marked performance improvements after sleep, whereas an equivalent period awake during the day does not provide significant off-line gains. Adapted from Gudberg et al. (13).

After initial encoding, memory traces undergo further processing, which takes place after we are no longer engaged with the learning task or environment. These off-line processes aid in stabilizing, and making more robust, learnt material (8–10). Depending on the type of input, this consolidation period may offer additional performance gains, specifically after a period of sleep, that are not due to further practice. For example, after practicing a new motor skill, such as a short, explicit motor sequence (Figure 1), young, neurologically intact individuals consistently show significantly improved performance on this task after sleep compared to an equivalent period of wakefulness (11–13).

Meanwhile, consolidation processes, including those involving sleep, may be more dynamic than previously thought, and there are a number of factors inherent in the learning context or task that impact on the expression and magnitude of the post-encoding consolidation gains observed with sleep. These findings may consequently have significant implications for the efficacy and long-term clinical outcomes of rehabilitative training paradigms.

### Consolidation Depends on Content and Context

A growing number of studies suggest that individual properties inherent to a given task may rely on different off-line processes of memory consolidation. For example, simply being aware of the underlying regularities or patterns in a motor learning task (explicit memory) has been shown to require specifically a period of sleep for observable off-line gains, whereas the same task performed without conscious awareness of such patterns (implicit memory) has been associated with significant improvements following the simple passage of time, and not specifically a period of sleep (12). That implicit learning may be time- rather than sleep-dependent is also consistent with recent findings (14, 15). Even different properties within the same task might be processed differently off-line. For instance, while the spatial or goal-based component of a motor task required a post-training consolidation period of sleep for performance gains, the movement property of the same task-related behavior relied specifically on wakefulness, and not the simple passage of time or sleep, to elicit off-line improvements (16). Similar findings have also been reported more recently (17), showing that a nap preferentially enhanced the allocentric (spatial) representation of motor learning but that, in contrast, the egocentric (motor) component of the task was only maintained, without further gains, regardless of whether the consolidation period involved sleep or wakefulness. Moreover, sleep has also been shown to increase the probability of extracting explicit rule-based information during an implicit task, such as might occur in moments of insight (18), suggesting that it is possible to transition at least from implicit to explicit memory.

Although the exact processes whereby memories are consolidated remain unclear, it may be appropriate to think of a given motor task as generally involving a variety of components, rather than being purely implicit or explicit in content, and that sleep may facilitate a restructuring of such information in the brain as reflected in altered task-related behavior.

#### Interference

**Figure 2:**
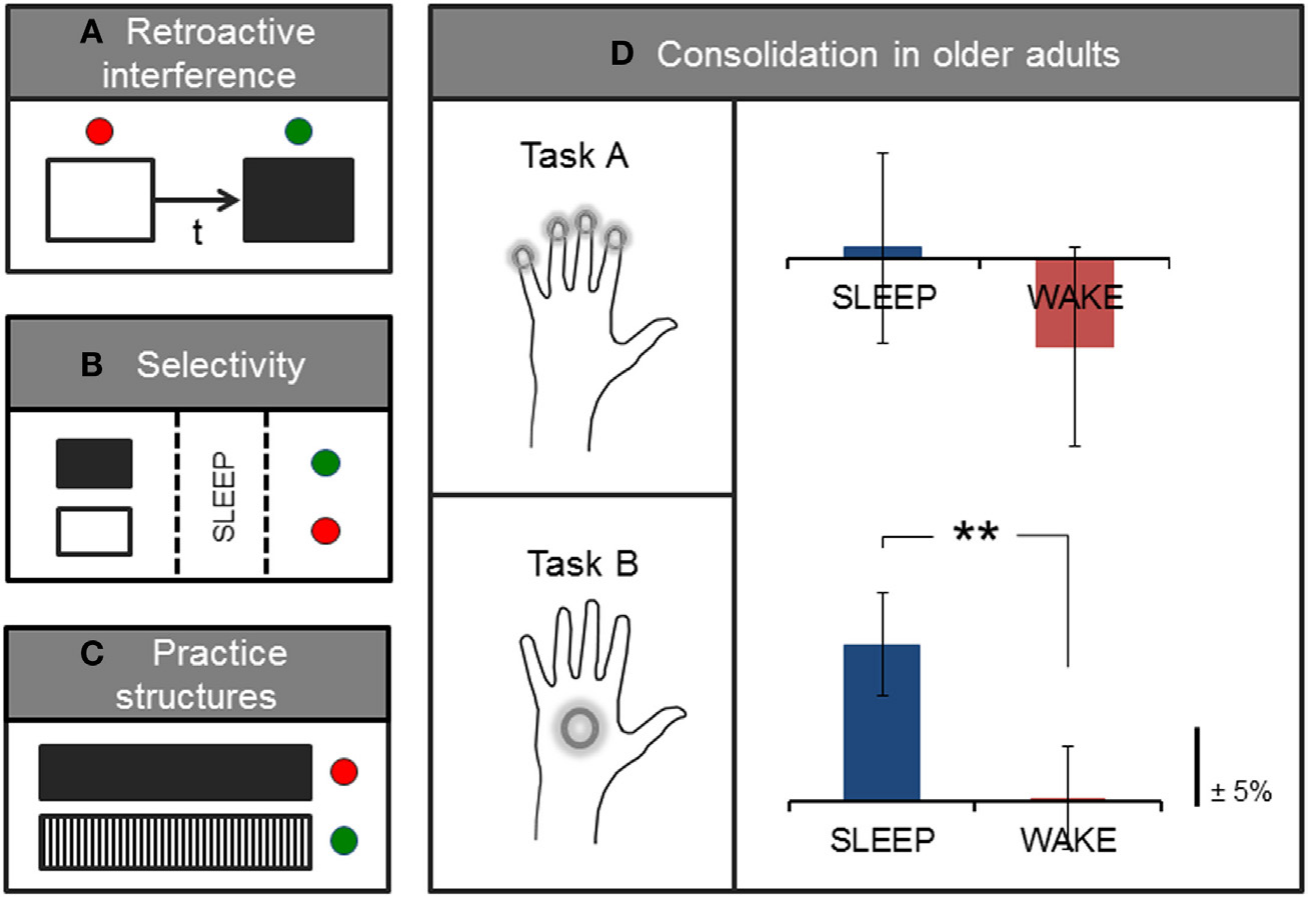
**Factors influencing or interacting with processes of consolidation**. Simplified sketch of specific variables that have been studied in the context of motor learning. **(A)** Pairing two motor tasks separated by a short time-interval (t) is associated with retroactive interference, and typical consolidation gains in performance may no longer be observed on the first task. **(B)** Expectation of future relevance or a monetary reward has been shown to elicit selective enhancement of specific (tagged, in black) memories during sleep. **(C)** Interleaved practice structures (black/white vertical) have also been associated with greater retention and transfer compared with blocked or massed practice (black solid). **(D)** On average, older adults show sleep-dependent motor memory consolidation on a whole-hand motor sequence task (Task B) but not on the same task requiring individuated finger movements to perform the sequence (Task A); adapted from (13). Green and red dots, respectively, denote the presence or absence of stabilization, improvement, or selective enhancement at retention testing.

Contrary to early accounts of consolidation that stipulated a more rigid form of memory storage, accumulating evidence is now suggesting that memories may undergo several time-sensitive stages of consolidation and reconsolidation. During this time, memory traces are highly labile and may once again be rendered susceptible to interference, even after initial memory consolidation has taken place (19–21). For example, behavioral interference of a motor memory may occur if an alternate motor learning task is ­introduced shortly after training on an initial motor task (Figure 2A). Here, studies have tended to investigate the effects of this type of (retroactive) interference using two similar tasks (e.g., sequence learning) paired at different time intervals. However, retroactive interference may occur even in the case of two different motor-based tasks if, for example, both tasks are performed with the same limb [(22); for an account of retroactive effects of declarative memory on motor learning, see Brown and Robertson (23)]. This effect is differentiated from the (related) concept of contextual interference, which is thought to result from variable or interleaved practice during acquisition training, and which may actually increase levels of retention [(24, 25); further discussed in Section “Practice Structures”]. Classic studies of consolidation and reconsolidation (e.g., 21, 26) have elegantly demonstrated this retroactive disruption to be specific to the consolidation process (rather than to performance at immediate retest, which was not reduced after the second, interference task) as well as specific to sleep (not wake) consolidation. Interestingly, this effect was only observed for the accuracy of the performed motor task, but not for the speed of execution (21). The time-window of potential interference may, however, be short-lived and may be suspended following initial stabilization (robustness to interference), which has been shown to take effect within a few hours proceeding initial training (21, 26, 27), and possibly earlier if a short nap follows initial practice (28). However, even after a period of sleep consolidation, an initially stable memory may be interrupted if the memory is reactivated (e.g., through a brief retest) immediately before training on another task (21). In other words, it is possible that merely reactivating an existing, stabilized motor memory facilitates a renewed labile period due to a process of destabilization, which under normal circumstances is usually followed by a form of reconsolidation or, if coupled with interference training, possibly degradation or complete extinction of the memory (21, 29). This dynamic consolidation process is likely a very useful mechanism of plasticity that allows the brain to continually revisit and update existing stored memories or representations in light of newly acquired information (20, 30). However, these findings may also have implications for the structure of learning paradigms in an applied setting, such as in the context of rehabilitation, particularly those involving training on tasks with different motor-based content in relatively close succession.

#### Selectivity

In addition to these time-sensitive phases, memory processing also appears to encompass highly selective mechanisms that have been shown to occur shortly after initial encoding as well as during subsequent sleep consolidation [(31, 32); Figure 2B]. For example, Wilhelm et al. (32) showed that different types of memories, including those formed during motor sequence learning, could be selectively enhanced during sleep if participants expected to be tested on the task at a later time, as compared with participants who did not expect to be retested on the task. Similar selective motor gains have been demonstrated for the anticipation of monetary reward at retest (31), and are consistent with selective reactivation processes thought to occur during sleep (33, 34). These intriguing findings, although preliminary, indicate that it may be possible to pre-select particular motor memories for further processing during sleep, which consequently show benefits over and above ‘untagged’ memories. However, further work that elucidates the underlying mechanisms of selective enhancement is needed, and it remains unclear whether sleep replay is a causal mechanism for the selective gains observed after sleep consolidation. In addition, directed selectivity in memory processing may be particularly useful to further study in the context of rehabilitative therapy as a possible adjunct aid for augmenting the effects of training, for instance, by integrating aspects of physical training with cognitive components of perceptual saliency.

#### Practice Structures

Like other forms of real-world learning, rehabilitative motor training arguably has (at least) two desirable learning goals: long-term retention and transfer to other contexts and tasks (7, 35). However, unlike training paradigms associated with retroactive interference, combining different tasks in specific practice structures may actually offer benefits to learning outcomes. For instance, variable or interleaved practice has been shown to facilitate both improved retention and transfer (24, 36, 37) relative to other types of practice (e.g., massed or blocked; Figure 2C). This practice-dependent effect was initially conceptualized as ‘contextual interference’ (38), and may tap into specific latent dynamics associated with the learning/performance distinction (39, 40). Here, evidence hints at a seemingly paradoxical relationship between in-session performance gains and post-encoding processes of consolidation and learning. That is, while training paradigms such as massed motor practice often show immediate performance effects in the form of better within-session acquisition, such paradigms may counter-intuitively be less conducive to long-lasting memory retention and transfer (7, 35, 37). By contrast, variable practice, which may elicit more modest performance gains during training, has instead been associated with better stabilization (41), retention, and transfer (24, 36, 37) at subsequent retest sessions. In other words, maximizing performance within the training session may not be a useful marker of eventual learning. This is consistent with previous work showing no or an inverse correlation between in-session training gains and subsequent sleep-dependent improvements in performance (13, 42). Such practice-dependent dynamics have been demonstrated for a wide range of tasks, including motor skill and imagery (36, 37), for verbal domains (38), and even for the retention of surgical technical skills (43), suggesting that there may be wider implications for learning in different contexts including, for instance, mixed-content rehabilitative training.

Recent evidence has also shown a potential role for sleep consolidation in the processing of, at least, motor imagery with variable practice (37), and preliminary neuroimaging findings have implicated different cortical brain areas in the retention processes respectively supporting variable and constant practice structures (41). However, it is unclear what mechanisms, particularly relating to sleep, support the effects associated with variable practice, or how subtle differences in the timing of “interference” tasks (e.g., blocked sequential versus interleaved trials) may elicit seemingly dissociable effects on consolidation outcomes. Further studies are needed to clarify these specific mechanisms and to assess possible avenues for beneficial application in the clinic.

### Mechanisms of Sleep Consolidation

Although the exact neural mechanisms of sleep that support the different phases and types of memory consolidation remain unclear, it is thought that these occur in part through fundamental regulatory processes that ensure cellular homeostasis and the integration of information in the brain. This may occur through processes of desaturation of synaptic potentiation that accumulates during active wakefulness (44, 45). This downscaling, in turn, is thought to promote increased functional efficiency and signal-to-noise ratio in neural environments, thereby supporting memory processing and information transfer in the brain (46, 47). Sleep slow wave activity is thought to be one of the candidate oscillatory mechanisms underlying these essential processes, which, in the human electroencephalogram recorded from the scalp, is reflected primarily in the low-frequency, high-amplitude activity (<4 Hz) characteristic of deeper stages of sleep. This activity is greatest in the early cycles of the night and decreases as sleep progresses, and is thought to be a marker of sleep need (48, 49). Moreover, studies have shown that markers of synaptic potentiation, for example, following motor learning, are associated with a subsequent increase in sleep slow wave activity in both animals (50) and humans (48, 49). Interestingly, findings suggest that local changes in synaptic density due to learning may also lead to site-specific increases in slow wave activity (51). However, in addition to these important neural regulatory processes, sleep may also occupy a more “active” role in memory consolidation.

According to the standard model of systems consolidation, sleep supports a process whereby memory traces are reprocessed and transferred across distributed networks via cortico-hippocampal projections (52, 53). For example, several studies have demonstrated reactivations of neural firing patterns during sleep that were initially activated during wakeful learning (54–57). Moreover, activity that is typically associated with increased arousal states, such as learning (58), has also been shown to be highly prevalent during sleep (59). The presence of “active” states during sleep is also consistent with neuroimaging findings showing sleep reactivation of the same brain areas that were engaged during initial motor skill encoding (60). Several electroencephalographic (EEG) signatures have been linked with these active consolidation processes during sleep, including high-frequency activity such as spindles and sharp-wave ripples, which are thought to be temporally coupled to the depolarizing phase of the sleep slow oscillation (61, 62). Spindle activity has also been associated more specifically with behavioral gains in motor sequence performance following sleep (63, 64).

Interestingly, recent studies have demonstrated that it may be possible to experimentally induce memory reactivation during sleep by, for instance, presenting a sensory cue that was initially introduced during skill acquisition. This pairing was subsequently associated with significant post-sleep performance gains (2, 65). Cued reactivation during sleep has also been linked with an increase in explicit knowledge (66). Moreover, applying transcranial direct current stimulation (tDCS) to the premotor cortex during sleep, using a paradigm thought to enhance excitability, was found to elicit a post-sleep improvement in the recall of the practiced motor sequence (67).

Collectively, there is growing evidence supporting a role of sleep in both up- and down-regulating the expression of plasticity-related activity in memory consolidation. Such plasticity-evoked regulations could map onto, or partially underlie, proposed sleep functions including “active” consolidation (e.g., through processes of synaptic potentiation or reactivation) and homeostatic regulatory processes (e.g., through mechanisms of synaptic desaturation). Meanwhile, how are motor memories affected when sleep changes significantly over time, such as with aging?

### Age-Related Changes

It is well established that older age is associated with changes in sleep architecture. Studies highlight a multiplicity of age-related variations [for meta-analysis, see Ohayon et al. (68)], including time spent in different sleep stages, such as increased stage 1 and 2 non-rapid-eye-movement (NREM) sleep, reduced slow wave sleep and overall sleep efficiency [including greater fragmentation (69, 70)], decreased incidence of sleep spindles (71, 72), as well as more indirect age-related differences in sleep processes in the form of altered circadian cycles, brain structure and function. For instance, neuroimaging evidence suggests that structural changes in the brain with older age, such as atrophy in the medial prefrontal cortex, could influence key sleep architecture, including non-REM slow waves (73). This is further supported by cross-sectional findings showing gradual decrements in k-complex morphology across the lifespan (74), as well as results suggesting a significant association between cortical atrophy and poor sleep quality (75).

Moreover, older age has also been associated with specific memory deficits related to the sleep-dependent motor gains typically observed in younger adults (76–78). It is possible that changes to sleep architecture with aging are responsible for such impairments in consolidation. However, it may also be that additional age-related factors contribute to observed deficits. For example, accumulating evidence suggests that fine motor ability deteriorates markedly with older age (79–82). Importantly, most tasks adopted to assess sleep-dependent motor consolidation in older age-groups have tended to rely entirely on fine motor skill, such as rapid individuated finger movements to execute a repeating number sequence (Figure 2D). We recently showed that subtle changes to individual task demands, which reduce this fine motor requirement, in turn, reveal significant sleep-dependent gains in older adults (13). Therefore, there may be multiple factors, for example, relating to motor control and sleep remodeling, that interact with consolidation processes in aging.

In the context of stroke rehabilitation, age-related changes in motor ability and sleep architecture could have important implications for physical rehabilitation in older patient groups and the efficacy of therapeutic training paradigms.

## Sleep after Stroke

The concepts of learning and consolidation explored here have further implications in clinical settings. For example, the role of sleep in consolidating motor memories may change dramatically after stroke-related brain damage, which in turn may have consequences for movement rehabilitation, which depends on motor learning and consolidation. In contrast to young, neurologically intact adults, who generally show sleep-dependent effects for explicit motor memories and time-dependent effects for implicit motor learning (12, 13), patients who had suffered a stroke required specifically a period of sleep to consolidate both implicit and explicit motor learning (83).

There are many possible reasons for altered consolidation of motor memories after stroke, including sleep disruption and altered learning processes. Sleep problems are highly prevalent following both ischemic and hemorrhagic stroke, affecting up to 78% of stroke patients (84). These include insomnia (85) and sleep-related breathing disturbances (86, 87), as well as daytime impairments associated with post-stroke fatigue (88). Sleep-related breathing disturbances, such as obstructive sleep apnea, are the most prevalent sleep disorders following stroke (84), and some form of sleep apnea may be present in as many as 50–70% of stroke survivors (87). Multiple direct and indirect mechanisms may underlie stroke-related sleep disorders, including changes to endogenous circadian control of essential sleep–wake functions, medication-dependent influences on sleep architecture, as well as increased daytime inactivity and napping. While sleep-related problems are commonly reported during the acute phase after stroke, there is also increasing evidence of disordered sleep–wake patterns in later, more chronic stages post-stroke. For example, in a cross-sectional sample of patients assessed 1 to 15 years following first-ever stroke, self-reported sleep quality was found to be relatively consistent regardless of time since stroke, whereas daytime sleepiness worsened with advanced chronicity (89). Such sleep–wake disruptions after stroke have, in turn, been linked with significant cognitive and attentional deficits (90, 91) as well as adverse health and clinical outcomes (87, 92–94). Therefore, it is likely that sleep-related problems following stroke may have an impact not only on processes of memory consolidation during sleep but also on the initial acquisition and encoding of new memories.

Moreover, both acute and chronic stage stroke have been associated with more specific imbalances in both the sleep and wake EEG relative to healthy controls. For instance, research suggests a higher prevalence of both slower and faster frequency oscillations (specifically in the delta, theta, and sigma ranges) during sleep at the infarct site following a stroke but also more widely across the affected hemisphere (95). In addition, an increase in slower frequencies have also been observed in the awake EEG of both acute and chronic stroke patients (95, 96), which may reflect an increased sleep need (97–99). This is consistent with common reports of daytime symptoms of sleepiness post-stroke (89, 95), although daytime sleepiness was not found to correlate with this low-frequency activity in the chronic phase post-stroke (96). Moreover, this slowing of neural activity during wakefulness is also in line with observed patterns of activity following sleep deprivation in non-clinical samples (100, 101) and has, in turn, been associated with severely reduced task performance as well as ability to form new memories (101–103). Reported increases in slow frequency activity during wakeful resting state after stroke may also be indicative of significant maladaptive responses after injury, and the up-regulation of wakeful delta activity has, for example, previously been associated with encephalopathy as well as structural brain changes such as reduced white matter volume (104, 105). Therefore, altered electrical activity in the brain following stroke may partly reflect processes of plasticity and homeostatic regulation, but also potentially more maladaptive local processes in response to brain damage.

Given the relatively wide-ranging influences that stroke may have on sleep microarchitecture, it is conceivable that such changes may have further impact on both skill acquisition and consolidation processes during physical recovery. Although evidence assessing the impact of sleep quality on clinical outcomes after stroke is scarce, a recent study in stroke survivors found that higher slow wave activity in the sleep EEG correlated with poorer functional recovery in the chronic stage of stroke (95). In another study, low subjective feelings of recovery post-stroke were correlated with poorer sleep scores on the Pittsburgh Sleep Quality Index (106). The link between sleep and stroke outcomes is consistent with preliminary research investigating ischemic stroke models in rats suggesting that sleep deprivation may have a significant detrimental effect on motor recovery (108). In humans, treatment of sleep problems such as obstructive sleep apnea with continuous positive airway pressure (CPAP) was associated with both improved motor recovery and reduced daytime sleepiness in stroke patients (107). However, further studies in human patient groups are needed to assess the relative contributions of sleep problems and microarchitecture on clinical outcomes after stroke rehabilitation.

During initial memory encoding, there is also evidence to suggest that the damaged brain may respond differently to the provision of explicit information in the context of motor training. For instance, retention on an implicit motor sequence task was impaired in stroke patients, but not healthy controls, when paired with explicit information about the underlying sequence (109, 110). Interference effects between declarative and procedural components during rehabilitative training have been associated with altered brain activity patterns in stroke patients (111). However, such task-related impairments may crucially depend on underlying patient-specific factors such as lesion site, severity, or time after stroke. For recovery more broadly, individual differences in brain and behavior may also significantly influence the long-term clinical efficacy achieved with rehabilitation training. Neuroimaging results further suggest that functional activation changes, particularly in the sensorimotor regions of the brain, after stroke rehabilitative therapy may be able to explain some of the observed variance in motor recovery (112). This is consistent with research in healthy adults, suggesting that individual differences in brain structure and function significantly predict differences in behavior and motor skill learning (113, 114). However, neither corticospinal tract integrity nor lesion volume predicted rehabilitation gains in motor performance in chronic stroke patients (115). Meanwhile, evidence suggests that time-dependent factors may interact with individual differences, with potential implications for motor recovery. For example, poorer clinical outcome scores in the early, but not late, stages post-stroke were associated with recruitment of brain areas including the contralesional cerebellum and ipsilesional premotor cortex during an isometric hand-grip task (116). Together, these findings highlight the importance of individual differences and temporal factors in recovery pathways. However, it is unknown how these factors influence, or interact with, altered sleep processes post-stroke.

In addition, it is important to note that sleep disorders may also pre-date stroke onset as both obstructive sleep apnea and habitual snoring have been attributed as independent risk factors in stroke pathology (117). Therefore, an important challenge for future studies will be to dissociate the specific, directional effects of stroke on sleep-related disorders and microarchitecture from any pre-existing conditions or sleep disorder. Evidence taking into account sleep disturbances following other types of brain damage, such as traumatic brain injury, may here provide useful clues to further dissociate specific mechanisms governing these relationships. Investigations of sleep and consolidation after stroke are further challenged by potential limiting factors and confounds, such as the relatively extensive list of post-stroke medications thought to influence sleep–wake patterns and specific sleep architecture, as well as difficulties in directly comparing between highly diverse patient groups, lesion site and volume, or time post-stroke. Further studies are needed that carefully address these challenges and to elucidate the extent to which variables that are shown to influence consolidation in healthy groups (e.g., interference, practice structures) translate to specific learning outcomes following brain damage.

### Practical Considerations

Sleep has been shown to benefit many processes of learning and memory, and may also have an important role in the homeostatic regulation of neural mechanisms. Meanwhile, brain damage such as stroke has been associated with a number of sleep–wake disorders, which in turn may have detrimental effects on both short- and long-term recovery. Therefore, integrating sleep assessments as a routine part of rehabilitative care is likely to have significant implications for stroke recovery and long-term disability.

Here, the availability of relatively cost-effective and non-invasive devices, such as actigraphy monitoring, may provide a practical solution for routine or longer-term sleep assessment. These can often be worn continuously throughout the day and night with minimal intrusion to the patient or treatment course. However, such devices provide only indirect measurements of sleep by analyzing activity-rest accelerometer data. For more direct and in-depth measurements of sleep, EEG remains the gold-standard technique, often as a part of full polysomnographic assessment. In addition, strategies should be considered that promote better sleep on the ward, such as strategies for noise reduction during sleep (118), ensuring chronobiologically appropriate light levels during the daytime and evening (119), and potential use of other approaches to improving sleep such as cognitive-behavioral and/or pharmacotherapy in stroke patients suffering from comorbid sleep–wake disturbances (117). Moreover, given the high prevalence of sleep-related breathing disturbances following stroke (87), a combination of routine monitoring (e.g., with respirography) and the provision of suitable treatment options (e.g., with CPAP) may here help to significantly improve both sleep and clinical outcomes of patients with moderate to severe sleep apnea (117). However, an ideal approach would consider these and other sleep-related factors within an integrated dynamic care pathway that is both tailored to the individual patient as well as appropriately updated throughout the treatment course. It will be useful to further consider whether such tailored stroke rehabilitation programs may help to alleviate individual symptoms and rebalance sleep–wake dynamics (e.g., by reducing daytime sleepiness or any disproportionate representation of low-frequency neural activity during wakefulness).

In the context of physical rehabilitation training, experimental evidence suggests that motor learning and retention may be sensitive to particular training structures, such as variable and massed practice. Here, sleep may also offer intermediate stabilization of new learning by further protecting against retroactive interference, and may even serve a more active role in consolidation by, for example, drawing on concepts of selectivity or cued reactivation during training and sleep. In addition to maximizing the quality of night-time sleep, the effects of encouraging naps following rehabilitation settings could be tested, given the beneficial effects of even brief periods of sleep following motor learning [e.g., (120)]. However, these effects have primarily been studied in non-clinical settings. It will, therefore, be important to determine how the variables of practice structure, sleep quality, and sleep timing interact with memory consolidation in the clinic, and whether there are stroke-specific factors that may influence these processes such as, for example, greater susceptibility to the influence of explicit information during implicit motor training.

## Conclusion

Converging evidence suggests that memory consolidation is dynamic and complex, capable of achieving various memory states, including stabilization, enhancement, reactivation, as well as reconsolidation over time. In addition to supporting these processes of memory formation, sleep is also a critical component for enabling the acquisition of new memories. This review has outlined a selection of candidate processes that have been explored in the sleep and motor learning literature, and which may have important implications for physical rehabilitation training. Stroke is here a useful model in which to situate such a discussion, however, it is important to bear in mind that different neurological conditions may have very different effects on sleep, learning, and consolidation. Moreover, while we have primarily focused on sleep consolidation in the context of motor-based learning, these concepts likely have broader application to other types of learning and rehabilitation. Further studies in clinical settings are needed to examine the role of sleep quality on rehabilitation training and whether the beneficial effects of sleep consolidation translate to meaningful clinical outcomes in rehabilitative care. However, increasingly incorporating sleep as an integral part of clinical assessments and training paradigms will undoubtedly have important implications for rehabilitation outcomes.

## Conflict of Interest Statement

The authors declare that the research was conducted in the absence of any commercial or financial relationships that could be construed as a potential conflict of interest.
